# Supplementary material for: Structural and evolutionary determinants of Argonaute function
Source: Nucleic Acids Res. 2025 Sep 29;53(18):gkaf962. doi: 10.1093/nar/gkaf962 (PMC12477606; doi:10.1093/nar/gkaf962)
Supplement: gkaf962_Supplemental_Files [file gkaf962_supplemental_files.zip › Supplementary_Figs.pdf]

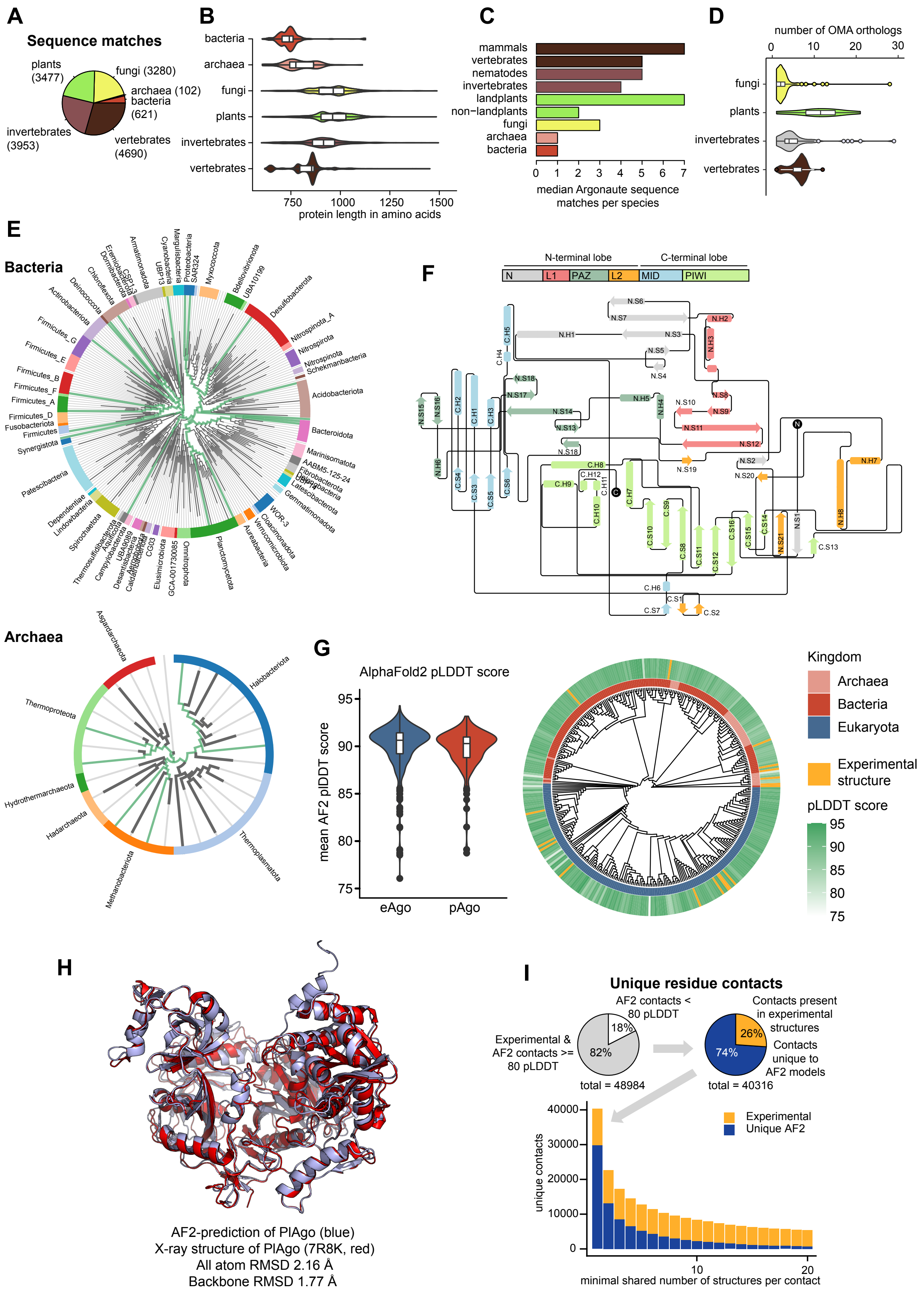

Supplementary Fig. S1

A Pie chart of phylogenetic origin of Argonaute sequences found in the UniprotKB database.
B Violin plots of length of putative Argonaute sequences by phylogenetic origin. We set a

cutoff at a length of 1500 amino acids for visualization purposes. **C** Median number of Argonautes per species found in this study. **D** Number of Argonaute proteins sourced from the OMA database across fungi, plants, invertebrates and vertebrates. **E** Bacterial and archaeal phylogenetic class annotation derived from the Genome Taxonomy Database (GTDB) database and visualized with AnnoTree. Taxonomies with pAgo representatives included in the reference alignment are highlighted in green. **F** Cartoon representation of the Argonaute topology with labeled helices and beta-strands. **G** Boxplot of mean full protein pLDDT scores from AF2 and projection on Argonaute dendrogram with experimental structures highlighted in yellow. **H** Structural overlay of AF2-predicted structural model of *Pseudooceanicola lipolyticus* Argonaute (PlAgo) and the published structure (PDB: 7R8K). **I** Full residue contact network from all available structures in this study. Contacts were only considered that were either from experimental structures or both contact partners had an AF2 pLDDT >= 80. Of the full set of contacts used in the study 26% can be found in at least one experimental structure. The proportion of contacts present in at least one experimental structure increases when filtering by the minimal shared number of contacts.

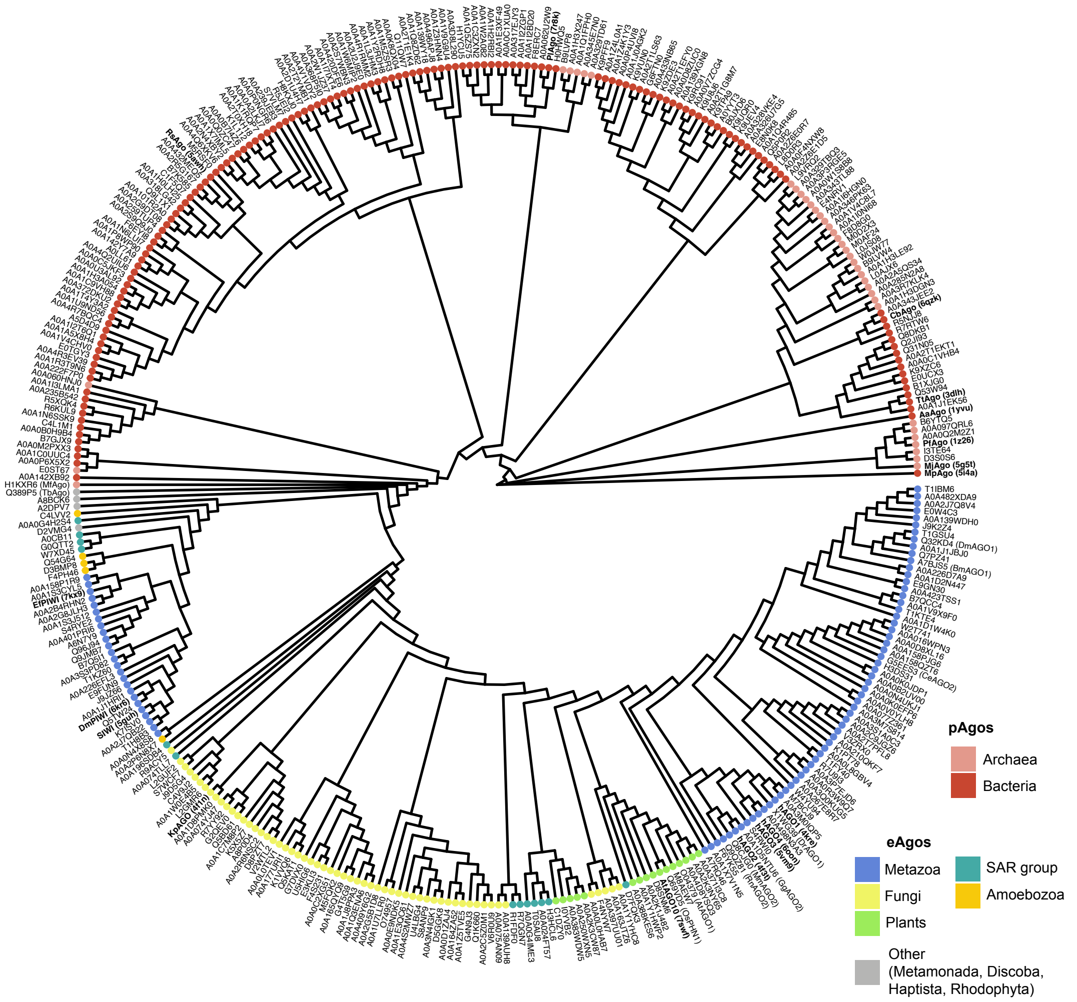

Supplementary Fig. S2

Detailed phylogenetic tree of all protein sequences used in this study. The nodes of the tree are color-coded by phylogenetic group and annotated with the UNIPROT codes. Proteins with available experimental structures are annotated in bold by name and PDB code of the published structure that was used for the structural alignment.

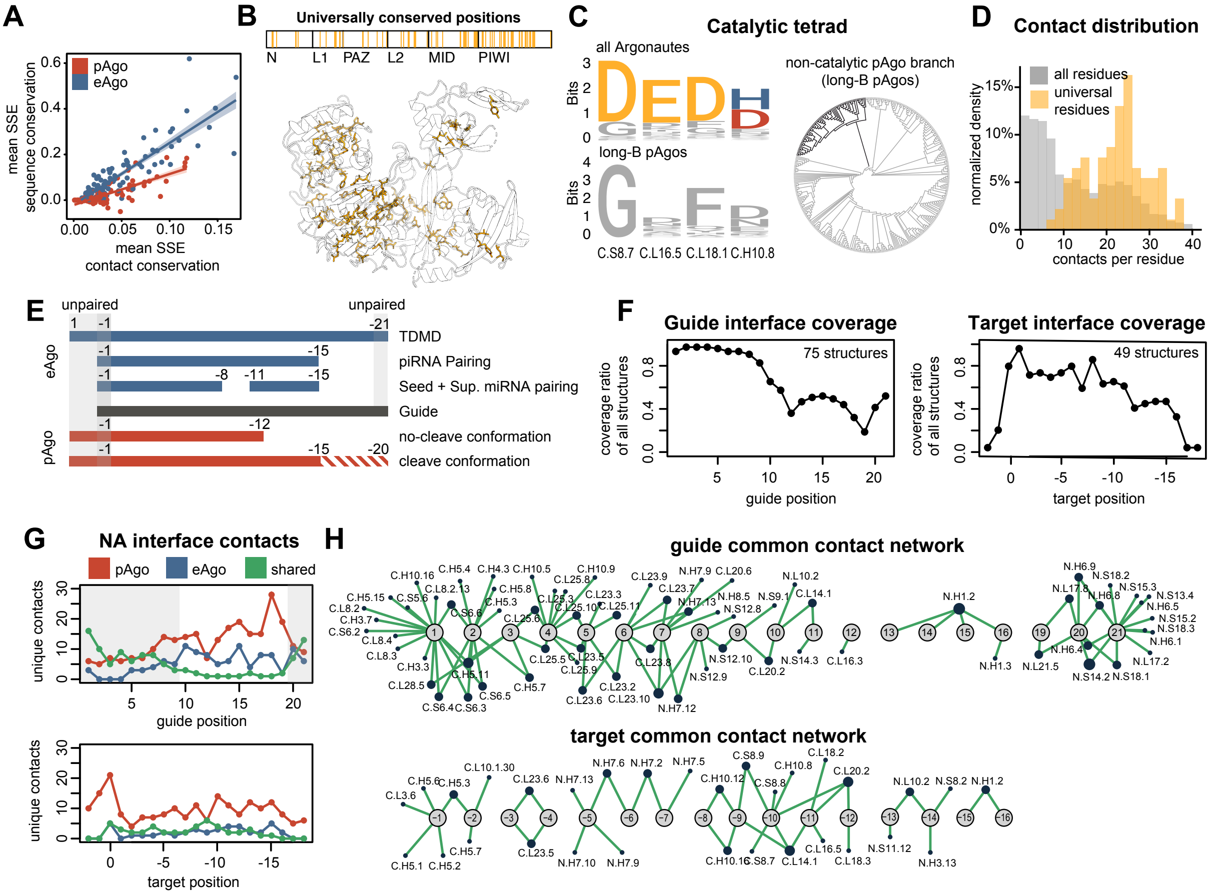

Supplementary Fig. S3

A Correlation plot of the mean contact and sequence conservation by SSE across all eAgos and pAgos. **B** Strip plot and cartoon structure representation of all universal signature positions. **C** Sequence logo plot of the catalytic tetrad for all Argonaute sequences and non-catalytic pAgos (long-B pAgos). Universal (yellow), pAgo (red) and eAgo (blue) -specific signature residues are highlighted. The long-B Ago branch is highlighted in the phylogenetic tree. **D** Histogram of contacts per position for all (grey) and universal signature residues (yellow) across all structures and models. **E** Guide and target numbering scheme employed in this study: Guide numbering 1-21 (for referencing of longer guides see Methods). The target was referenced to its complementary guide positions (e.g. target position -5 is complementary to guide position 5, positive target positions are unmatched 3' target ends). **F** Coverage of each guide and target position in all available structures that include either guide or both guide and target chains. **G** Total number of unique shared (green) and eAgo (blue) or pAgo (red) -specific contacts resolved by guide (top) and target (bottom) position. Guide contacts are predominantly shared in the seed and 3' end binding region (highlighted in grey). **H** Network representation of all shared guide (top) and target (bottom) contacts.

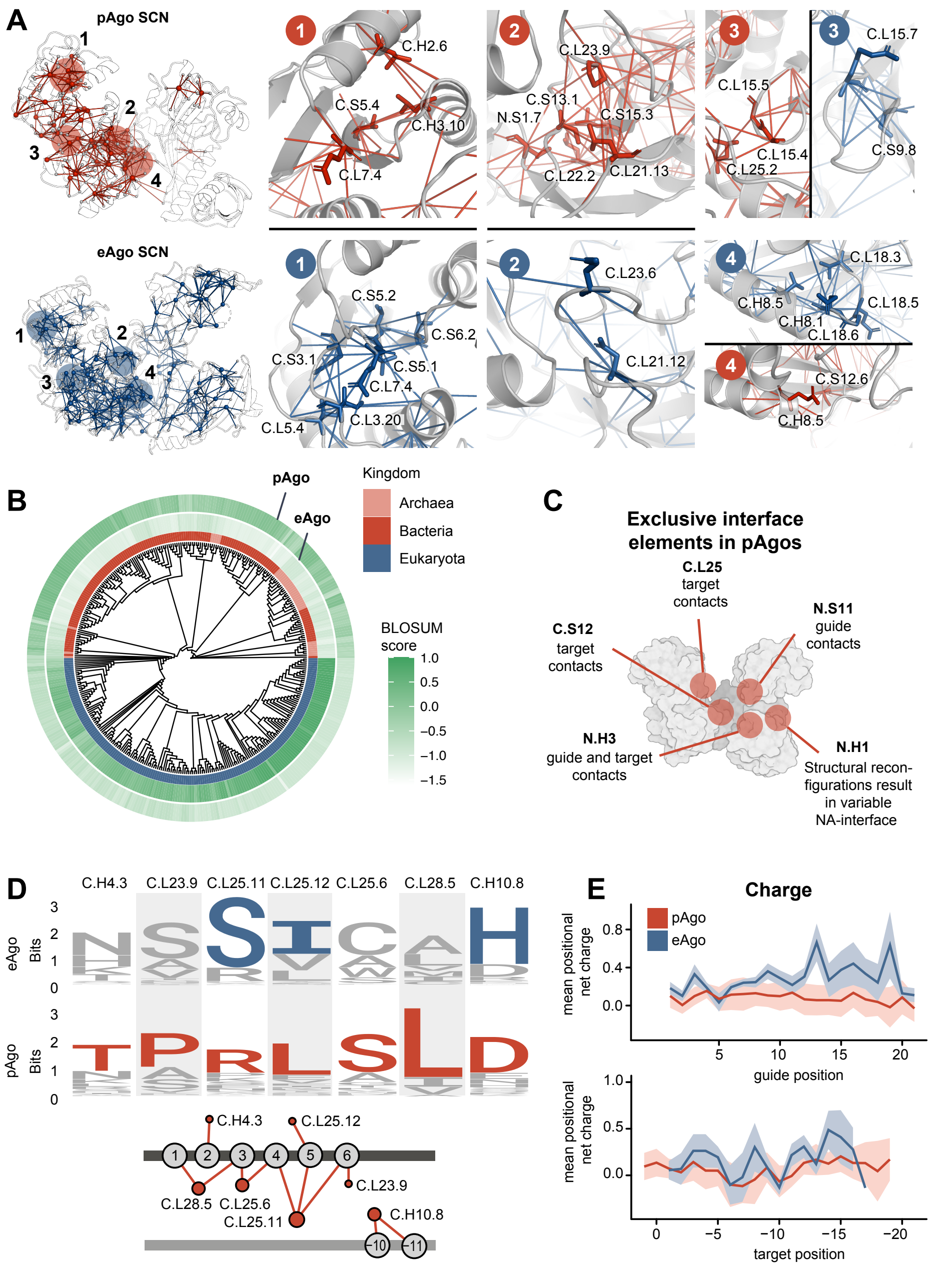

Supplementary Fig. S4

A Structural overlay of the pAgo and eAgo SCNs with numbered and highlighted regions in red (TtAgo, PDB: 3DLB) and blue (hAGO2, PDB: 4OLB), respectively. In the MID domain, pAgos show additional distinctly conserved contacts of signature residues in SSEs that form the 5' binding pocket (C.H2, C.H3), while a cluster of eAgo signature residues can be found in the connecting loops nearby (C.L5, C.L3, C.L7, panel 1). A similar cluster can be found in pAgos mediating contacts underneath the C.L23 loop, which directly contacts the guide in most Argonautes and only carries a few eAgo signature positions in the same region (panel 2). The structural reorientation of the loop C.L15 represents another conserved change from pAgos to eAgos and may imply that these features emerged to enable a new function in eAgos (panel 4). Features like the eAgo cs7 insertion element are also represented in our analysis (panel 5), which lead to a tugged-in conformation of the C.L18 loop, while the same loop is free to engage with the guide or target in pAgos. **B** Normalized BLOSUM score for eAgo and pAgo signature positions against all proteins included in this study with values mapped onto the phylogenetic tree as a heatmap. **C** Examples of exclusive NA interacting features found in pAgos. All NA contact data can be found in Supplementary Data 5. **D**

pAgo-specific signatures of the guide interface illustrated in the logo-plot and NA contact network representation. **E** Mean positional net charge of all positions resolved by guide/target position.

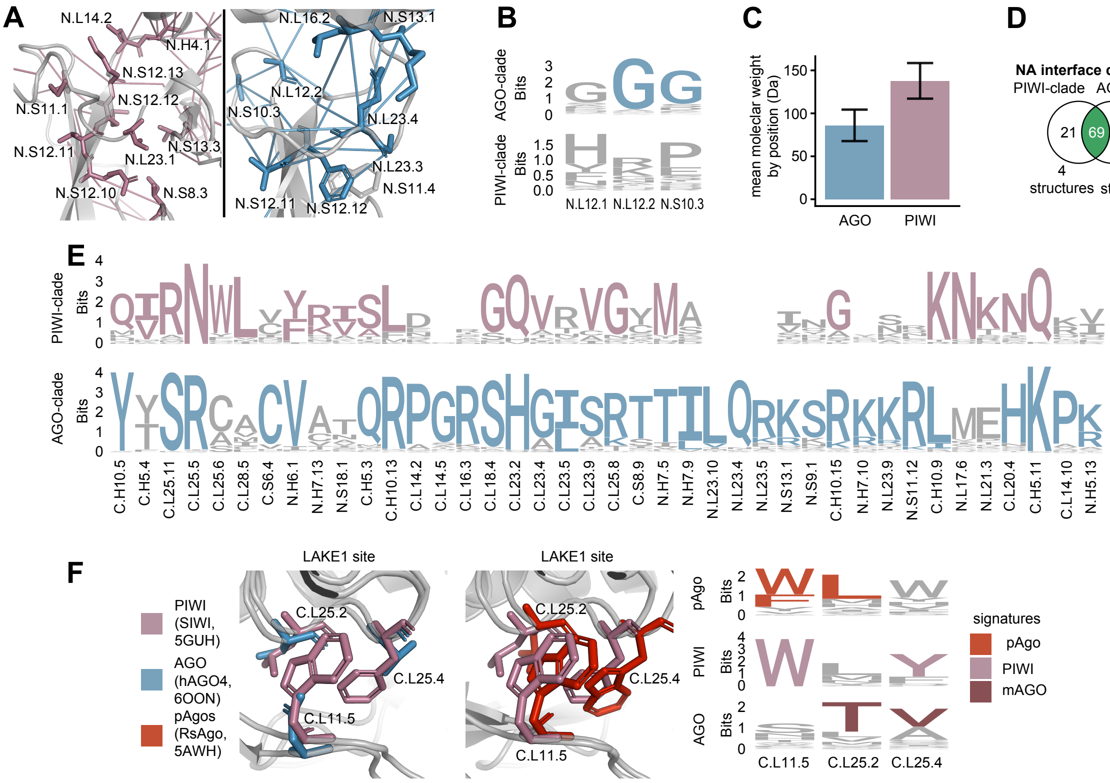

Supplementary Fig. S5

A The interface between the PAZ domain and the L2 stalk is mediated by distinct signatures in the AGO and PIWI-clade. The straight PAZ orientation of the AGO-clade is supported by an extended N.L23 interface (e.g. N.L23.4) and the glycine-rich loop N.L12. In AGO, N.S12.12 stabilizes the stalk twist by aromatic interactions with the universal residue N.S11.4. However, in PIWI the tilted PAZ conformation is stabilized by signature positions at the end of the N.S12 strand and N.L14 loop. **B** The N.L12 loop carries bulky residues that further preclude the straight PAZ conformation. Comparison of N.L12 loop residues between AGO and PIWI clade. The positions in the loop are conserved as glycine in the AGO-clade. In PIWI, the loop is not conserved and instead is composed of bulky residues. **C** Mean molecular weight of the positions in **B** for PIWI and AGO. **D** Total NA interface contacts from the available structures in the PDB separated by clade (see Supplementary Data 5 for full annotation of each contact) **E** Sequence logo plot of all PIWI and AGO signature NA-contact positions. **F** Comparison of LAKE1 region in a PIWI, AGO and pAgo representative together with sequence logo plot highlighting signature positions.

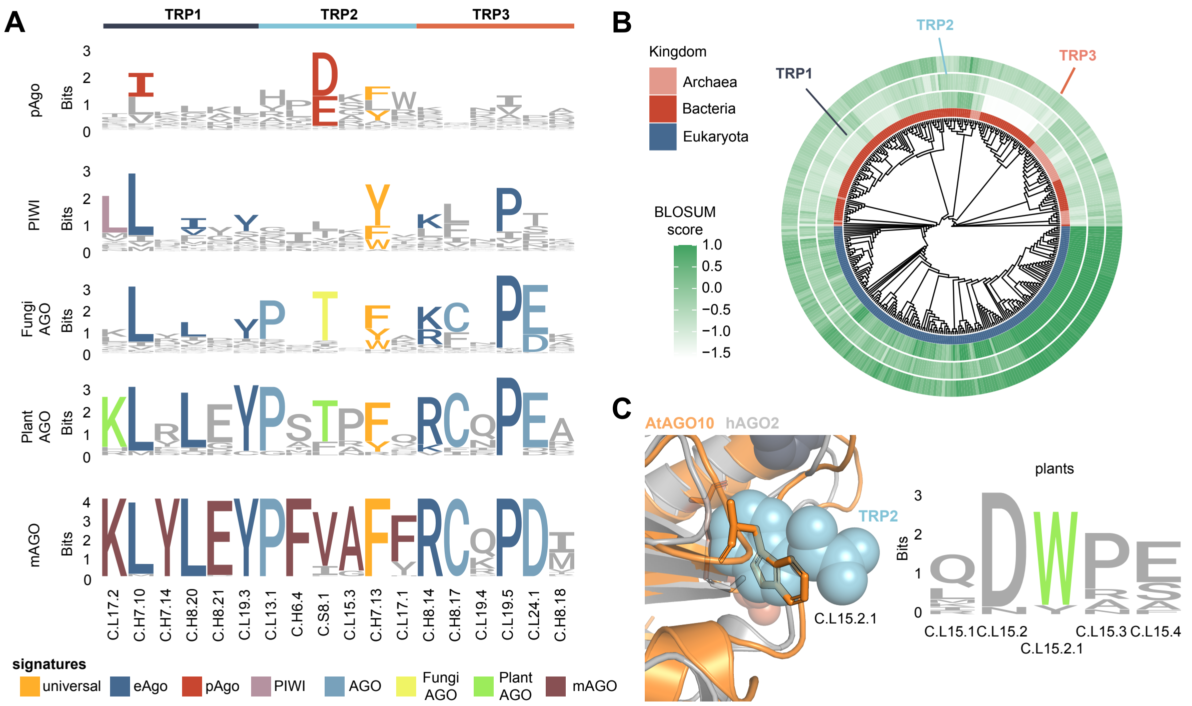

Supplementary Fig. S6

A Logo plot of the Argonaute GW-interface positions for multiple distinct phylogenetic groups annotated with signature positions. **B** Normalized BLOSUM score for mAGO consensus GW-interface positions against all proteins included in this study. **C** Structural overlay of AtAGO10 (orange, PDB:7SWF) and hAGO2 (grey, PDB:6CBD) at the TRP2 binding pocket. The C.L15.2.1 (732) position is localized in the GW pocket in AtAGO10. The logo plot of C.L15.2.1 and surrounding positions shows it to be highly conserved as tryptophan in plants.

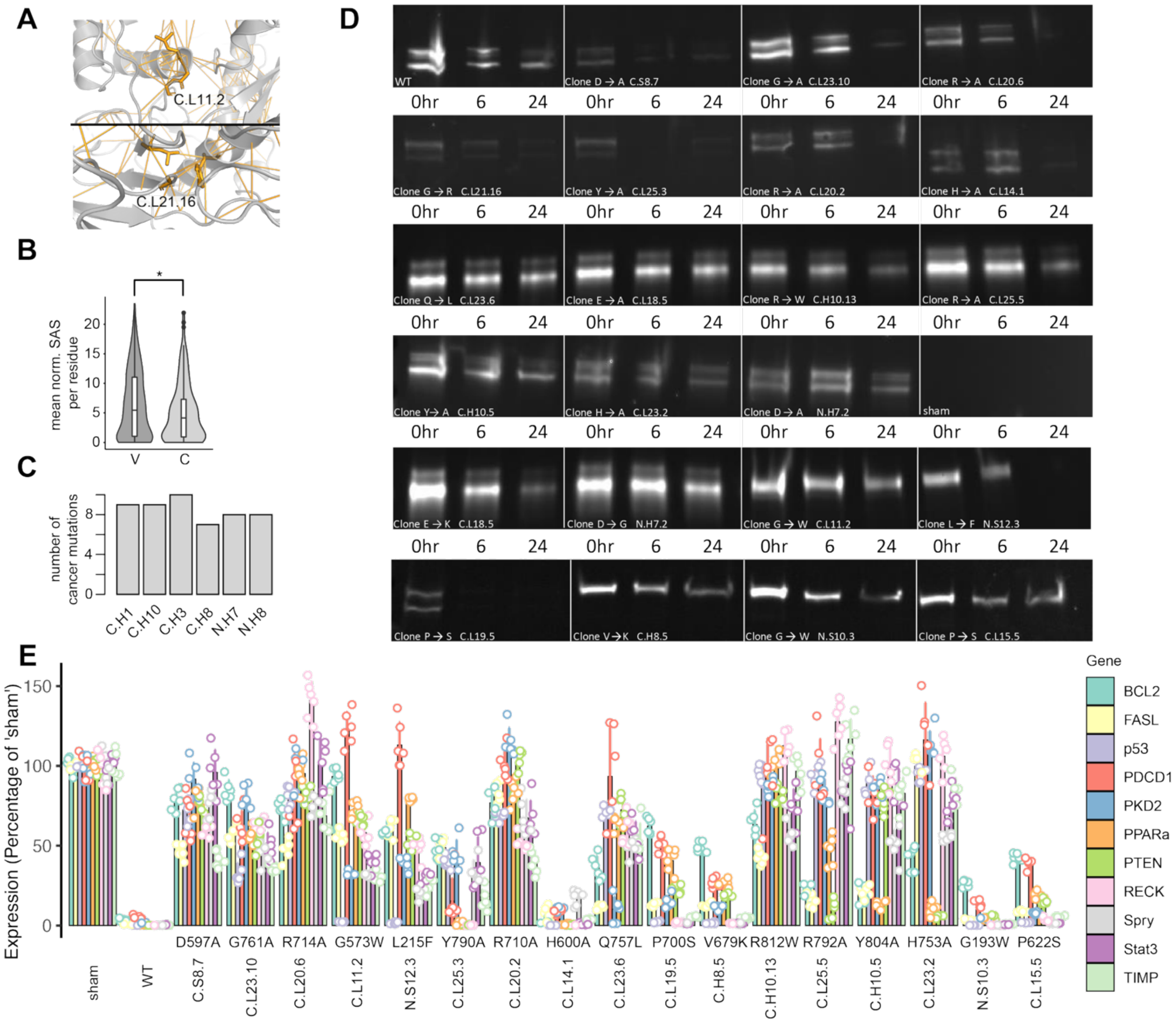

Supplementary Fig. S7

A Structural position within the universal SCN of two LESKRES glycine missense mutations. **B** Mean solvent accessible surface (SAS) for variant (V) and cancer (C) mutations. **C** Number of cancer mutations across the six most mutated SSEs **D** Western blotting of CHX-assay for AGO2 stability in hAGO2-GFP mutants. **E** RT-QPCR analysis of relative miR-21 target genes abundance following mutant hAGO2/miR-21 co-transfection.

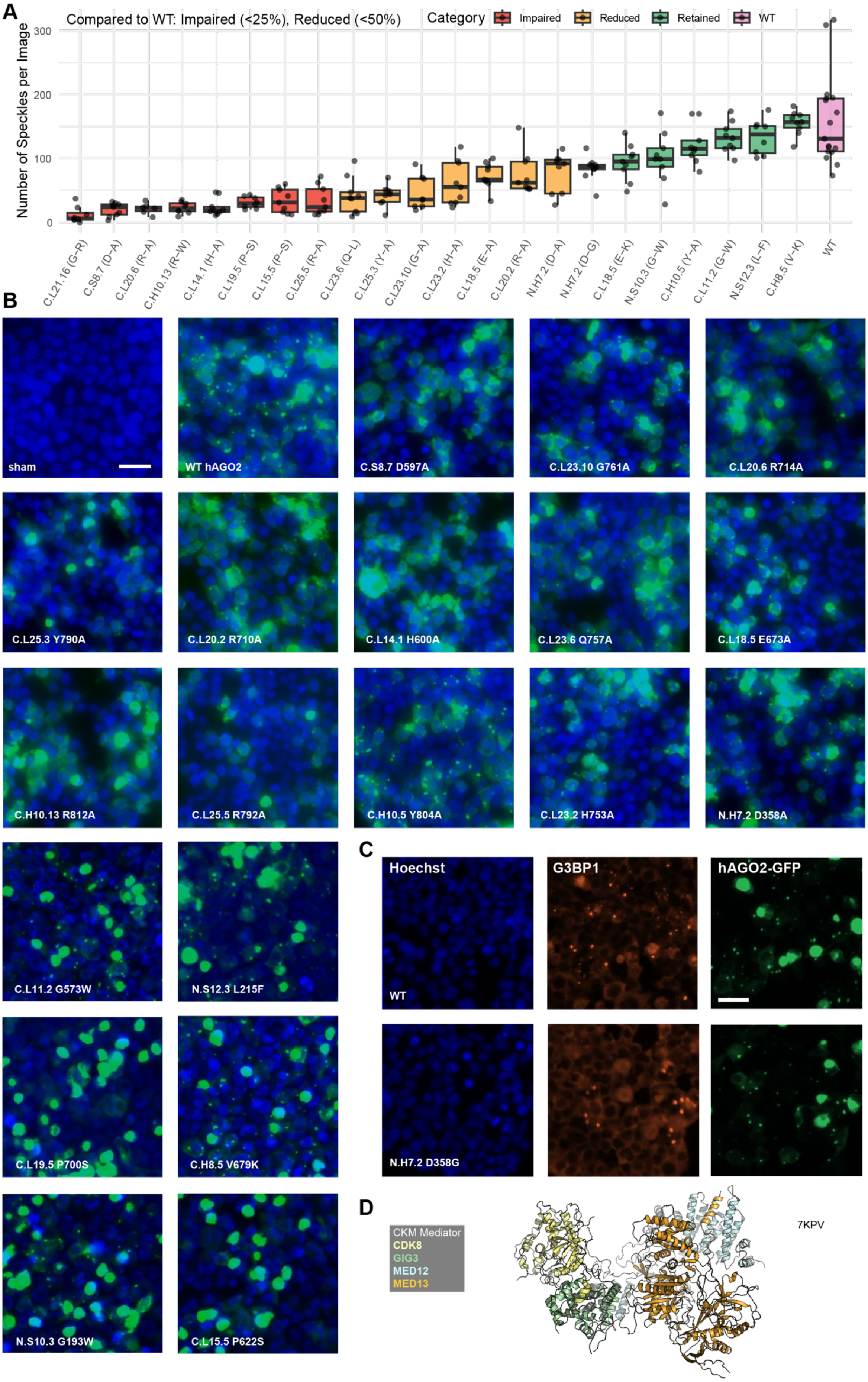

A
Compared to WT: Impaired (<25%), Reduced (<50%)
Category Impaired Reduced Retained WT
Number of Speckles per Image
0
100
200
300
C.L21.16 (G–R)
C.S8.7 (D–A)
C.L20.6 (R–A)
C.H10.13 (R–W)
C.L14.1 (H–A)
C.L19.5 (P–S)
C.L15.5 (P–S)
C.L25.5 (R–A)
C.L23.6 (Q–L)
C.L25.3 (Y–A)
C.L23.10 (G–A)
C.L23.2 (H–A)
C.L18.5 (E–A)
C.L20.2 (R–A)
N.H7.2 (D–A)
N.H7.2 (D–G)
C.L18.5 (E–K)
N.S10.3 (G–W)
C.H10.5 (Y–A)
C.L11.2 (G–W)
N.S12.3 (L–F)
C.H8.5 (V–K)
WT
B
sham
WT hAGO2
C.S8.7 D597A
C.L23.10 G761A
C.L20.6 R714A
C.L25.3 Y790A
C.L20.2 R710A
C.L14.1 H600A
C.L23.6 Q757A
C.L18.5 E673A
C.H10.13 R812A
C.L25.5 R792A
C.H10.5 Y804A
C.L23.2 H753A
N.H7.2 D358A
C.L11.2 G573W
N.S12.3 L215F
C.L19.5 P700S
C.H8.5 V679K
N.S10.3 G193W
C.L15.5 P622S
C
Hoechst
G3BP1
hAGO2-GFP
WT
N.H7.2 D358G
D
CKM Mediator
CDK8
GIG3
MED12
MED13
7KPV

Supplementary Fig. S8

A Number of speckles per image for wild-type (WT) and indicated mutants. Mutants are categorized based on significant reduction in speckle formation compared to WT (Wilcoxon test, Benjamini-Hochberg adjusted p-values < 0.05) and the extent of reduction: Impaired (<25% of WT mean), Partially Impaired (<50% of WT mean), or Retained. Boxes represent interquartile range (IQR), lines are medians, and dots are individual images. **B** Live cell imaging of hAGO2-GFP (green) following Arsenite exposure. Nuclear signal (blue) overlain. Scale bar 20µm. **C** Immunostaining of G3BP1 (red) in hAGO2-GFP (green) transfected HEK293 cells, demonstrating stress granule (small speckles) formation following Arsenite exposure, with nuclear signal (blue). Scale bar 20µm. **D** Structure of yeast CKM Mediator complex with annotated subcomponents (PDB: 7KPV).
