# Supplementary material for: Structural and evolutionary determinants of Argonaute function
Source: Nucleic Acids Res. 2025 Sep 29;53(18):gkaf962. doi: 10.1093/nar/gkaf962 (PMC12477606; doi:10.1093/nar/gkaf962)
Supplement: gkaf962_Supplemental_Files [file gkaf962_supplemental_files.zip › Sup. Data.zip!/Sup. Data/sequences_alignments_tree/interactive_topology_lookup.html]

Argonaute Topology Lookup


# Argonaute Topology Lookup Tool

This tool allows you to look up UniProtIDs, topology codes and amino acid information from the Argonaute alignment data.

1. Load Data File

Please upload the **Supplementary Data 2.xlsx** file.


2. Query Data

A. Find by AA Position

Enter UniProtID (partial or full):

Enter Amino Acid Number:

Search

B. Find by Topology Code

Enter UniProtID (partial or full):

Enter Argonaute Topology Code:

Search

Search Results
Clear Inputs & Results

#### Topology & Sequence

**Topology Code:**

**Amino Acid:**

**Position in Protein:**

#### Protein Details

**UniProt ID:**

**Gene Names:**

**Organism:**

**Organism ID:**

**Domain:**


Search Proteins by Taxonomy or Species

Enter Taxonomy Keyword:

Search Proteins
Clear

### Matching Proteins:

|
|  |
